# Supplementary material for: Probing the Conformational States of Thimet Oligopeptidase in Solution
Source: Int J Mol Sci. 2022 Jun 30;23(13):7297. doi: 10.3390/ijms23137297 (PMC9266445; doi:10.3390/ijms23137297)
Supplement: Supplementary file 1 [file ijms-23-07297-s001.zip › ijms-1761733-supplementary.pdf]

Supplementary material

Probing the Conformational States of Thimet Oligopeptidase in Solution

Marcelo F.M. Marcondes[1,2], Gabriel S. Santos[1], Fellipe Bronze[1], Mauricio F.M. Machado[1], Kátia R. Perez[1], Renske Hesselink[2], Marcel P. de Vries[3], Jaap Broos[2]* and Vitor Oliveira[1]*

Content

Table S1. 2
Figure S1. 3
Figure S2. 5
Figure S3. 6
Figure S4 7
Figure S5. 8
Figure S6 9
Modeling of Thimet oligopeptidase (TOP) in its “closed” state 10
Swiss-Model report 10
Swiss-Model Methodology 12
References cited in the Swiss-model methodology 14
Swiss-model References 14

Table S1. Activity of WT TOP and TOP mutants in hydrolyzing the substrate QFS.

Sample	*Relative Rate*
WT TOP	1.00
TOP W124F	1.31
TOP W355F	0.50
TOP W390F	0.12
TOP W614F	2.37
TOP W511F/W513F	0.50

The rates of peptide hydrolysis, relative to the rate found for WT TOP, were determined at 37°C in 50 mM Tris-HCl buffer, pH7.4, containing 100 mM NaCl and 0.5 mM of DTT. Based in the kinetic parameters determined for the WT TOP we fixed a substrate concentration of 10 x K_M (~Vmax conditions [QFS] = 50µM).

Figure S1.

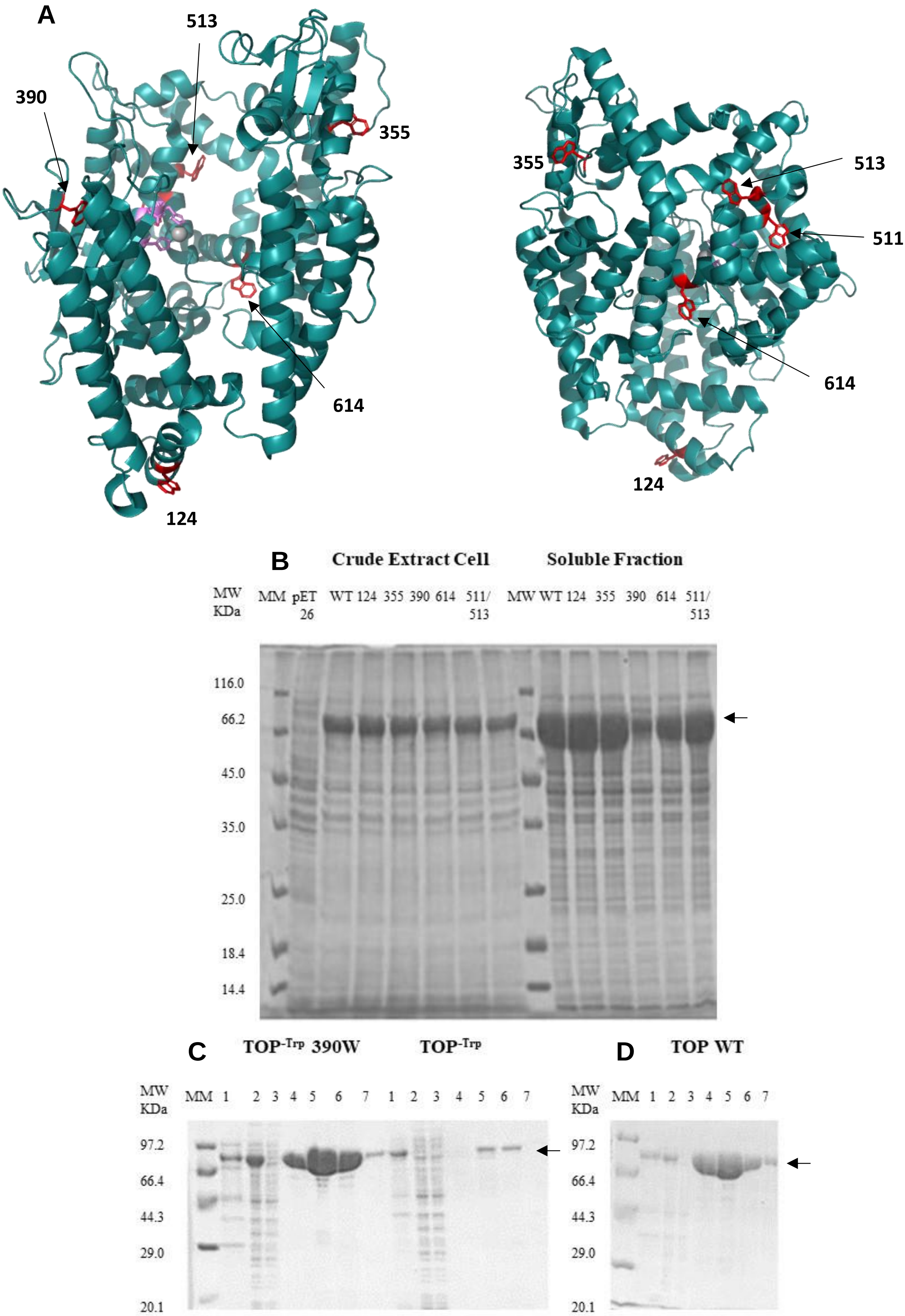
A
513
390
355
614
124
355
513
511
614
124
B
Crude Extract Cell
Soluble Fraction
MW KDa
MM pET 26
WT 124 355 390 614 511/ 513
MW WT 124 355 390 614 511/ 513
116.0
66.2
45.0
35.0
25.0
18.4
14.4
C
TOP-Trp 390W
TOP-Trp
MW KDa
MM 1 2 3 4 5 6 7 1 2 3 4 5 6 7
97.2
66.4
44.3
29.0
20.1
D
TOP WT
MW KDa
MM 1 2 3 4 5 6 7
97.2
66.4
44.3
29.0
20.1

Figure S1. Residue tryptophan 390 is essential for expressing TOP as soluble protein. **Panel A -** Ribbon representation of the TOP crystal structure (PDB 1S4B) at two different views (rotations), chosen to best show all Trp residues present on the structure. The tryptophan side chain residues are shown in red. The active site zinc ion is the grey sphere. The side chain of 3 residues (His^{473}, Glu^{474} and His^{477}) that bind the zinc ion are also shown (in purple). (Figures were made with Swiss pdb Viewer v.4.1.0). **Panel** B - WT TOP and TOP mutant expression experiments – SDS PAGE gel loaded with *E. coli* cell lysate and soluble fractions of lysed cells (supernatant after centrifugation at 27,000g, 20 min). Lanes: pET26 – empty vector (negative control); WT – TOP wild type; 124 - mutant TOPW124F; 355 - mutant TOPW355F; 390: mutant TOPW390F; 614: mutant TOPW614F and 511/513 - double mutant TOPW511/W513F. **Panel C -** SDS PAGE gel of the single-Trp mutant TOP^{-Trp}W390and mutant TOP^{-Trp} during different stages of its purification. Lanes: 1 - crude cell lysate; 2 - soluble fraction (supernatant after centrifugation at 27,000g); 3 – flow through (non-bound fraction) of the Ni-NTA column; 4, 5, 6 and 7 (eluted fractions 1-4, respectively). **Panel D** – fractions 1-7 collected during the elution of WT TOP with 150 mM imidazole. The arrow is at the expected position of TOP.

Figure S2. JA-2 structure

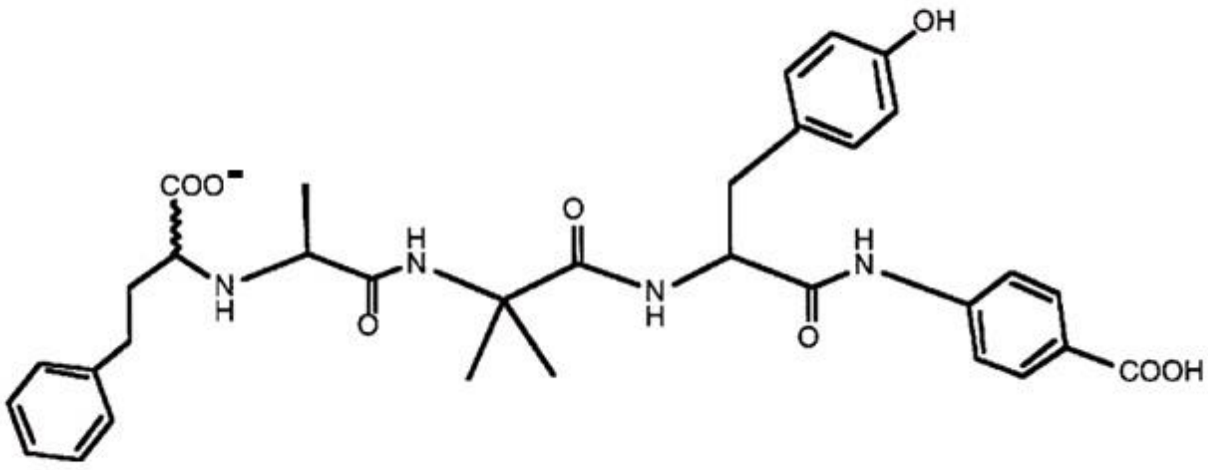

Figure S2. JA-2 structure. JA-2 - *N*-[1-(*R*,*S*)-carboxy-3-phenilpropyl]-Ala-Aib-Tyr-*p*-aminobenzoate). The carboxyl group of the *N*-[1-(*R*,*S*)-carboxy-3-phenilpropyl] moiety is thought to bind the catalytic zinc ion of TOP.

Figure S3.

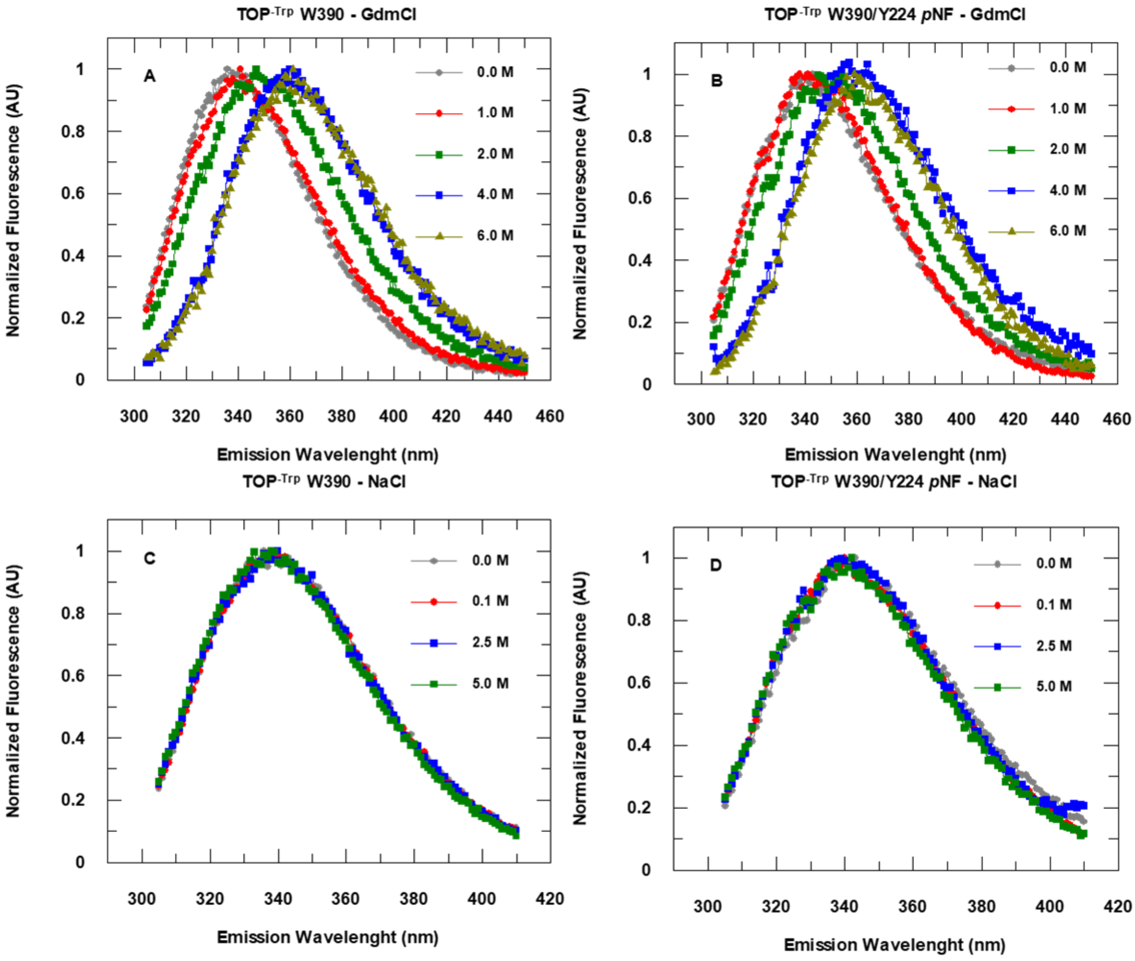

Figure S3. Emission spectra of TOP^{-Trp} W390 and TOP^{-Trp} W390/Y224 *p*NF mutants recorded at increasing salt concentrations: Figures A and B – Emission spectra recorded at different concentrations of GdmCl; Figures C and D – Emission spectra recorded at different concentrations of NaCl.

Figure S4

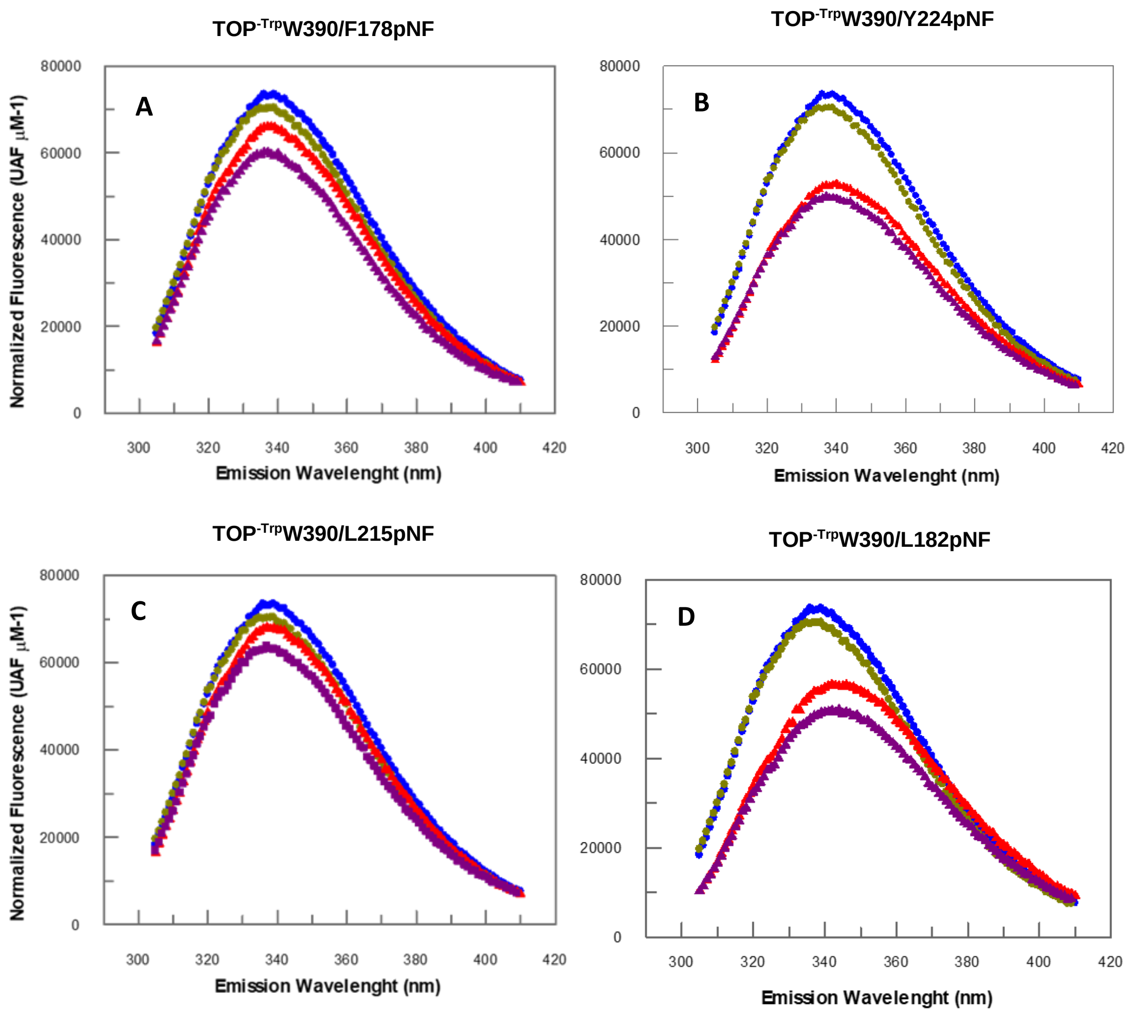

Figure S4. Fluorescence emission spectra of FRET experiments between Trp390 residue and *p*NF placed at different positions in the TOP structure in the absence and presence of inhibitor JA-2. Emission spectra of the single Trp residue TOP mutant, TOP^{-Trp}W390, in the absence (blue line) or in the presence of JA-2 (green line). Emission spectra of the double labeled TOP mutants, in the absence (red line) or in the presence of JA-2 (purple line): Panel A - TOP-TrpW390/F178pNF; Panel B - TOP-TrpW390/Y224pNF; Panel C - TOP-TrpW390/L215pNF; Panel D - TOP-TrpW390/L182pNF.

Figure S5.

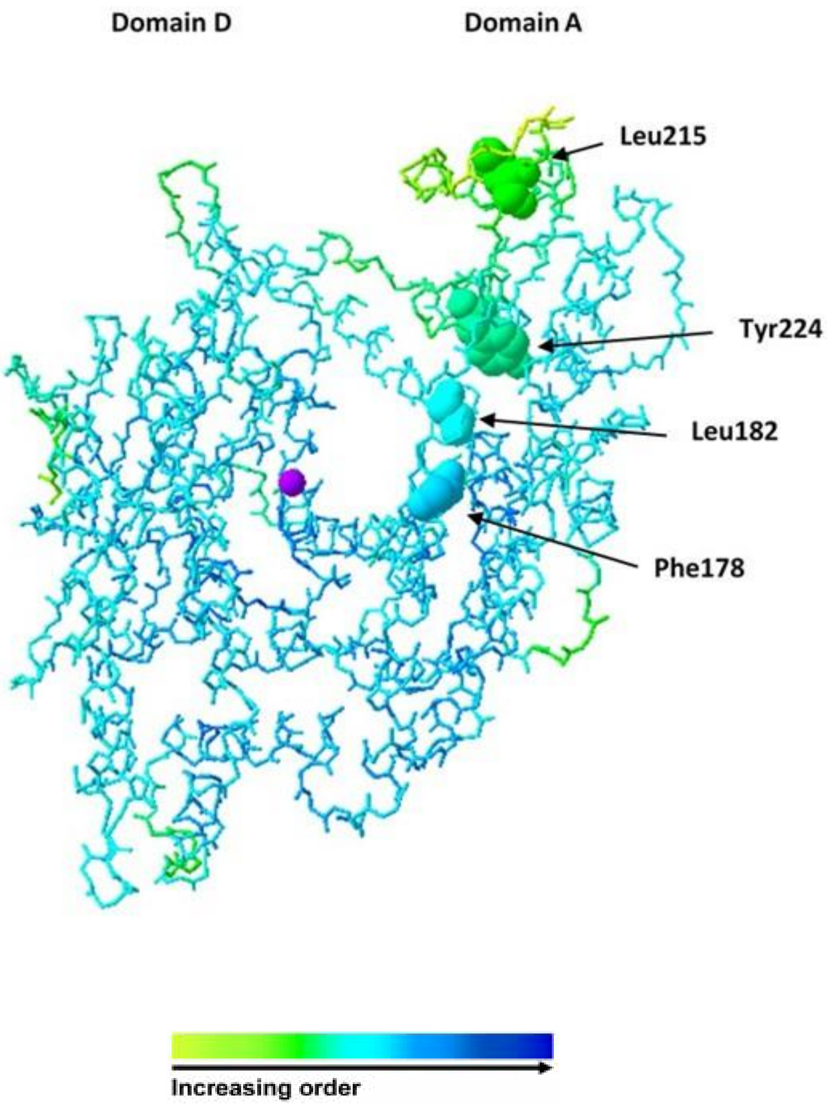

Figure S5 – Different positions of *p*NF in the TOP structure. Main chain representation of the TOP structure (PDB 1S4B) with the side chains of the residues chosen to be replaced by the *p*NF at each TOP mutant. A colored scale is used to represent differences in the temperature B-factor. Note that residues Leu215 and Tyr224 are in green to yellow regions, which are potentially more flexible while residues Phe178 and Leu182 are in blue regions, which are more rigid. The zinc ion is represented as a pink sphere. Figure made by using Swiss pdb Viewer v.4.1.0.

Figure S6 – **Time resolved fluorescene anisotropy decay of TOP^{-Trp} W390 labeled with 5-fluorotryptohan.**

TOP^{-Trp} W390 was labeled with 5-fluorotryptohan (5-FW) as described (JBC (2010) 285, 25324-25331) because specific excitation of the Trp^{390} position is challenged by the presence of 26 Tyr residues in TOP^{-Trp} W390. 5FW labeled TOP^{-Trp} W390 (4.8 µM) was dissolved in 50 mM Tris HCl pH= 7.4, 30 mM NaCl and 0.5 mM DDT and the time-resolved fluorescence decay at 20 ^{o}C was measured using a TCSPC fluorescence set up described in detail before (JBC (2010) 285, 25324-25331). Excitation was at 304 nm. Data was analyzed using the TRFA data processing package, version 1.2, of SSTC, Belarusian State University, Belarus. The fluorescence decay analysis yielded 2 lifetimes of 3.4 ns (12%) and 4.6 ns (84%) (χ^2 = 1.097). The anisotropy decay could be fitted with a model of two exponentials, yielding θ_1 = 1.54 ns (β= 0.011) and θ_2 = 79 ns (β= 0.09) (χ^2 = 1.061). We note that the intrinsic anisotropy of 5-FW under these conditions is 0.25 (JACS (2004) 126, 22-23) indicating fast depolarization processes are taking place within the time-resolution of the set- up (~50 ps).

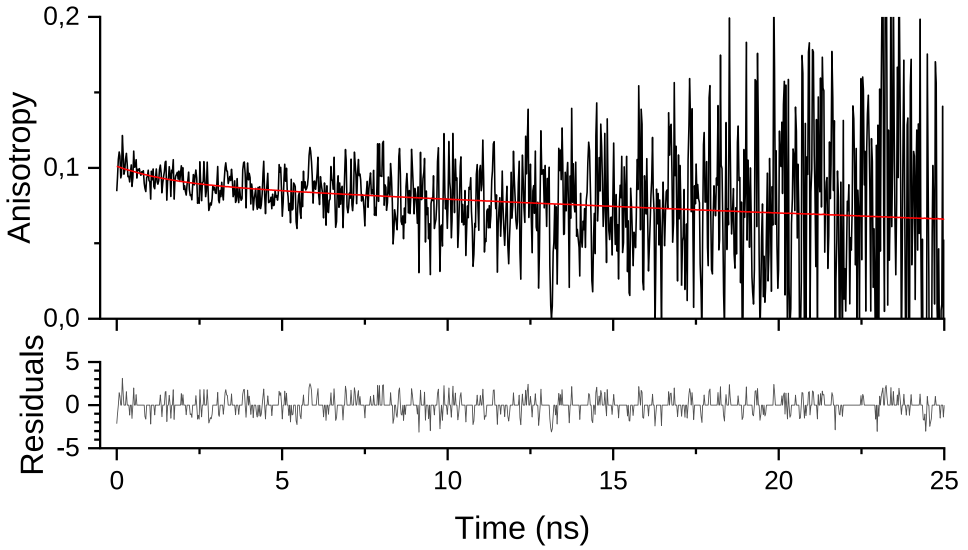

Modeling of Thimet oligopeptidase (TOP) in its “closed” state

Thimet oligopeptidase “closed” model was created using the Swiss-Model workspace and based on the crystallographic structure of the human neurolysin E475Q mutant (pdb 5LUZ) uploaded as a user-provided template.

The following details of the model quality and methodology were provided by the Swiss-Model workspace.

Swiss-Model report

Model #01	File	Built with	Oligo-State	Ligands	GMQE	QMEAN
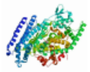	PDB	ProMod3 Version 1.1.0.	monomer	1 x ZN: ZN;	0.81	-0.60

Template	Seq Identity	Oligo-state	Found by	Method	Resolution	Seq Similarity	Range	Coverage	Description
template_upload.1.B	64.22	monomer	BLAST	Unknown	NA	0.49	21 - 677	0.95	Polypeptide

Ligand	Added to Model	Description
ZN	✓	ZN
ALA	✕ - Binding site not conserved.	ALA
CL	✕ - Binding site not conserved.	CL
CL	✕ - Binding site not conserved.	CL
GOL	✕ - Binding site not conserved.	GOL
HIS	✕ - Not biologically relevant.	HIS
LYS	✕ - Binding site not conserved.	LYS-PRO-ARG-ARG-PRO
PRO	✕ - Binding site not conserved.	PRO
PRO	✕ - Binding site not conserved.	PRO-ARG-ARG-PRO
TYR	✕ - Binding site not conserved.	TYR-ILE-LEU
TYR	✕ - Binding site not conserved.	TYR-ILE-LEU
ZN	✕ - Binding site not conserved.	ZN

QMEAN	-0.60
Cβ	0.22
All Atom	1.50
Solvation	1.97
Torsion	-1.04

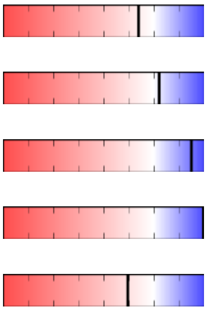

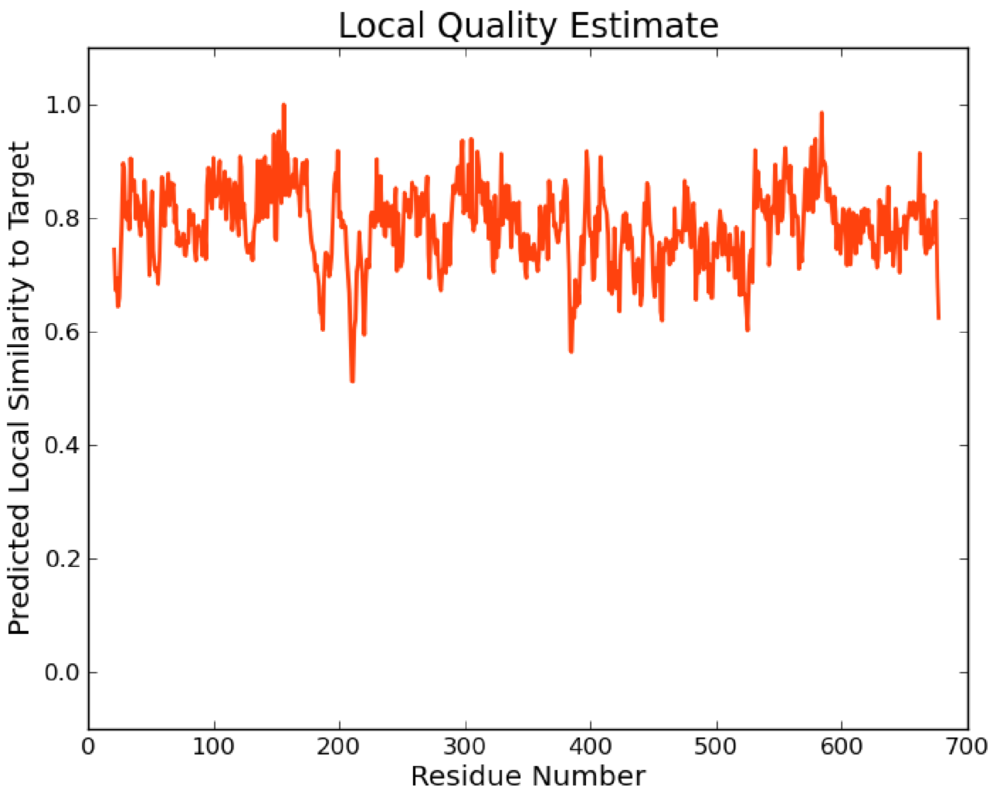
Local Quality Estimate
Predicted Local Similarity to Target
Residue Number
1.0
0.8
0.6
0.4
0.2
0.0
0
100
200
300
400
500
600
700

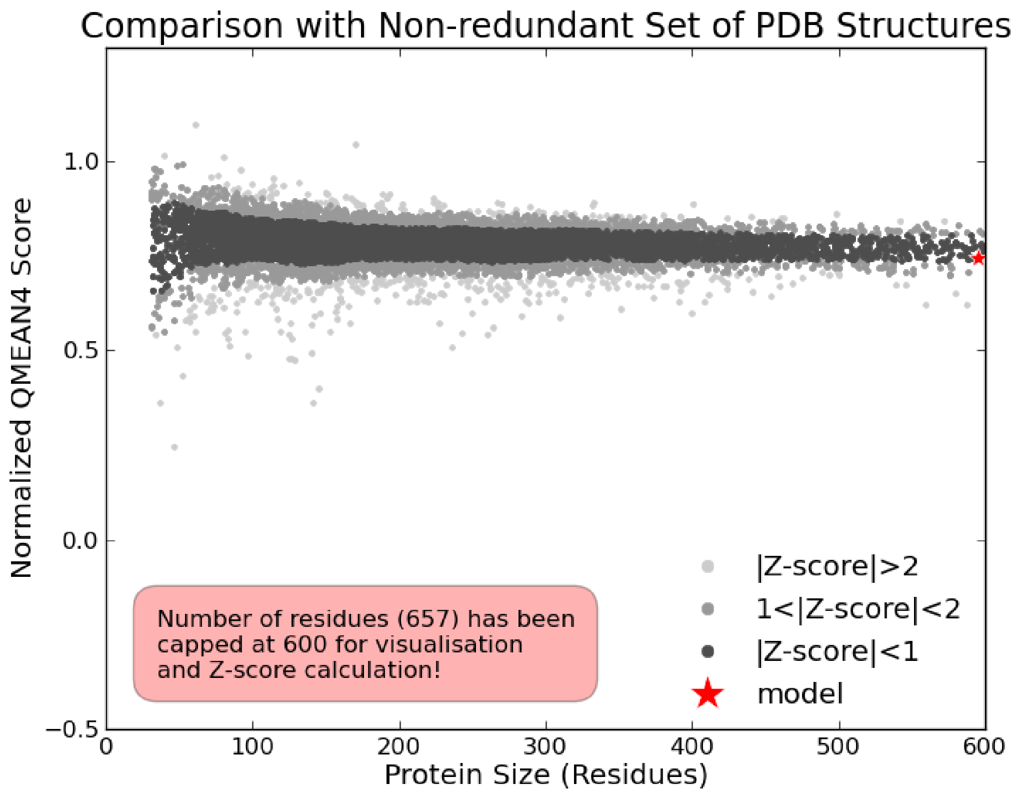
Comparison with Non-redundant Set of PDB Structures
Normalized QMEAN4 Score
Protein Size (Residues)
1.0
0.5
0.0
−0.5
0
100
200
300
400
500
600
|Z-score|>2
1<|Z-score|<2
|Z-score|<1
model
Number of residues (657) has been capped at 600 for visualisation and Z-score calculation!

Sequence alignment (target – rat TOP sequence / template - human neurolysin sequence)

```
Target                   KPPAACAGDVVDTVSPCSTVNHLRWDLSAQQIRALTTQLIEQTKCVYDRVGAQDF
template_upload.1.B      --------------------NVLRWDLSPEQIKTRTEELIVQTKQVYDAVGMLGI

Target                   EDVSYESTLKALADVEVTYTVQRNILDFPQHVSPNKDIRAASTEADKKLSEFDVE
template_upload.1.B      EEVTYENCLQALADVEVKYIVERTMLDFPQHVSSDKEVRAASTEADKRLSRFDIE

Target                   MSMRQDVYQRVVWLQEKIPKDSLKPEAARYLERLIKLGRRNGLHLPQDTQEKIKN
template_upload.1.B      MSMRGDIFERIVHLQETCDLGKIKPEARRYLEKSIKMGKRNGLHLPEQVQNEIKS

Target                   IKKRLSLLCIDFNKNLNEDTTFLPFTREELGGLPEDFLNSLEKTEDGKLKVTLKY
template_upload.1.B      MKKRMSELCIDFNKNLNEDDTFLVFSKAELGALPDDFIDSLEKTDDDKYKITLKY

Target                   PHYFPLLKKCHVPETRRLLEEAFNCRCKEENCAILKELVSLRAQKSNLLGFRTHA
template_upload.1.B      PHYFPVMKKCCIPETRRRMEMAFNTRCKEENTIILQQLLPLRTKVAKLLGYSTHA

Target                   DYVLEMNMAKTSQTVATFLDELARKLKPLGEQERAVILELKEAESAKRGLPFDGR
template_upload.1.B      DFVLEMNTAKSTSRVTAFLDDLSQKLKPLGEAEREFILNLKKKECKDRGFEYDGK

Target                   IHAWDMRYYMNQVEEDSYRVDQNLLKEYFPMQVVTRGLLAIYQELLGLTFTLEEG
template_upload.1.B      INAWDLYYYMTQTEELKYSIDQEFLKEYFPIEVVTEGLLNTYQELLGLSFEQMTD

Target                   AAAWHEDVRLYSVRDAASGEEIGKFYLDLYPREGKYGHAACFGLQPGCLRQDGSR
template_upload.1.B      AHVWNKSVTLYTVKDKATGEVLGQFYLDLYPREGKYNHAACFGLQPGCLLPDGSR

Target                   QLAIAAMVANFTKPTPDVPSLLQHDEVETYFHEFGHVMHQLCSQAEFAMFSGTHV
template_upload.1.B      MMAVAALVVNFSQGR---PSLLRHDEVRTYFHQFGHVMHQICAQTDFARFSGTNV

Target                   ERDFVEAPSQMLENWVWEKEPLMRMSQHYRTGGEAPEDLLEKLIKSRQANAGLFN
template_upload.1.B      ETDFVEVPSQMLENWVWDVDSLRRLSKHYKDGSPIADDLLEKLVASRLVNTGLLT

Target                   LRQIVLAKVDQVLHTQTDVDPAEEYARLCQEILGVPATPGTNMPATFGHLAGGYD
template_upload.1.B      LRQIVLSKVDQSLHTNTSLDAASEYAKYCSEILGVAATPGTNMPATFGHLAGGYD

Target                   AQYYGYLWSEVYSMDMFHTRFKQEGVLSPKVGMDYRTSILRPGGSEDASTMLKQF
template_upload.1.B      GQYYGYLWSEVFSMDMFYSCFKKEGIMNPEVGMKYRNLILKPGGSLDGMDMLHNF

Target                   LGRDPKQDAFLLSKGLQVEGCEPPAC
template_upload.1.B      LKREPNQKAFLMSRGLH---------
```

Below is the methodology presented from the downloaded report generated by the Swiss-Model workspace.

Swiss-Model Methodology

Model Building

Models are built based on the target-template alignment using ProMod3. Coordinates which are conserved between the target and the template are copied from the template to the model. Insertions and deletions are remodelled using a fragment library. Side chains are then rebuilt. Finally,

the geometry of the resulting model is regularized by using a force field. In the case loop modelling with ProMod3 fails, an alternative model is built with PROMOD-II (Guex, et al., 1997).

Model Quality Estimation

The global and per-residue model quality has been assessed using the QMEAN scoring function (Benkert, et al., 2011). For improved performance, weights of the individual QMEAN terms have been trained specifically for SWISS-MODEL.

Ligand Modelling

Ligands present in the template structure are transferred by homology to the model when the following criteria are met: (a) The ligands are annotated as biologically relevant in the template library, (b) the ligand is in contact with the model, (c) the ligand is not clashing with the protein, (d) the residues in contact with the ligand are conserved between the target and the template. If any of these four criteria is not satisfied, a certain ligand will not be included in the model. The model summary includes information on why and which ligand has not been included.

Oligomeric State Conservation

Homo-oligomeric structure of the target protein is predicted based on the analysis of pairwise interfaces of the identified template structures. For each relevant interface between polypeptide chains (interfaces with more than 10 residue-residue interactions), the QscoreOligomer (Mariani et al., 2011) is predicted from features such as similarity to target and frequency of observing this interface in the identified templates (Kiefer, Bertoni, Biasini, to be published). The prediction is performed with a random forest regressor using these features as input parameters to predict the probability of conservation for each interface. The QscoreOligomer of the whole complex is then calculated as the weight-averaged QscoreOligomer of the interfaces. The oligomeric state of the target is predicted to be the same as in the template when QscoreOligomer is predicted to be higher or equal to 0.5.

References cited in the Swiss-model methodology

- Altschul, S.F., Madden, T.L., Schaffer, A.A., Zhang, J., Zhang, Z., Miller, W. and Lipman, D.J. (1997) Gapped BLAST and PSI-BLAST: a new generation of protein database search programs. Nucleic Acids Res, 25, 3389-3402.

- Remmert, M., Biegert, A., Hauser, A. and Soding, J. (2012) HHblits: lightning-fast iterative protein sequence searching by HMM-HMM alignment. Nat Methods, 9, 173-175.

- Guex, N. and Peitsch, M.C. (1997) SWISS-MODEL and the Swiss-PdbViewer: an environment for comparative protein modeling. Electrophoresis, 18, 2714-2723.

- Sali, A. and Blundell, T.L. (1993) Comparative protein modelling by satisfaction of spatial restraints. J Mol Biol, 234, 779-815.

- Benkert, P., Biasini, M. and Schwede, T. (2011) Toward the estimation of the absolute quality of individual protein structure models. Bioinformatics, 27, 343-350.

- Mariani, V., Kiefer, F., Schmidt, T., Haas, J. and Schwede, T. (2011) Assessment of template based protein structure predictions in CASP9. Proteins, 79 Suppl 10, 37-58.

Swiss-model References

- Marco Biasini; Stefan Bienert; Andrew Waterhouse; Konstantin Arnold; Gabriel Studer; Tobias Schmidt; Florian Kiefer; Tiziano Gallo Cassarino; Martino Bertoni; Lorenza Bordoli; Torsten Schwede. (2014). SWISS-MODEL: modelling protein tertiary and quaternary structure using evolutionary information. Nucleic Acids Research (1 July 2014) 42 (W1): W252-W258; doi: 10.1093/nar/gku340.

- Arnold, K., Bordoli, L., Kopp, J. and Schwede, T. (2006) The SWISS-MODEL workspace: a web-based environment for protein structure homology modelling. Bioinformatics, 22, 195-201.

- Benkert, P., Biasini, M. and Schwede, T. (2011) Toward the estimation of the absolute quality of individual protein structure models. Bioinformatics, 27, 343-350
